# Ernie-Switcher: RNA foundation model-guided design of programmable RNA devices in a navigable landscape

**DOI:** 10.1093/bioinformatics/btag774

**Published:** 2026-10-26

**Authors:** Xin Lei, Runting Li, Yuzhong Liu, Jiesheng Huang, Yicheng Yang, Ruyuan Li, Shimin Liu, Pengxia Wang, Huaiguang Jiang, Wei Xiong

**Affiliations:** School of Future Technology, South China University of Technology, 777 Xingye Avenue East, Panyu District, Guangzhou, Guangdong, 511442, China; Guangdong Engineering Research Center of Low-Carbon Synthetic Biotechnology, South China University of Technology, 382 Waihuan East Road, Panyu District, Guangzhou, Guangdong, 510006, China; School of Future Technology, South China University of Technology, 777 Xingye Avenue East, Panyu District, Guangzhou, Guangdong, 511442, China; Guangdong Engineering Research Center of Low-Carbon Synthetic Biotechnology, South China University of Technology, 382 Waihuan East Road, Panyu District, Guangzhou, Guangdong, 510006, China; School of Biology and Biological Engineering, South China University of Technology, 382 Waihuan East Road, Panyu District, Guangzhou, Guangdong, 510006, China; School of Future Technology, South China University of Technology, 777 Xingye Avenue East, Panyu District, Guangzhou, Guangdong, 511442, China; Guangdong Engineering Research Center of Low-Carbon Synthetic Biotechnology, South China University of Technology, 382 Waihuan East Road, Panyu District, Guangzhou, Guangdong, 510006, China; Guangdong Engineering Research Center of Low-Carbon Synthetic Biotechnology, South China University of Technology, 382 Waihuan East Road, Panyu District, Guangzhou, Guangdong, 510006, China; School of Biology and Biological Engineering, South China University of Technology, 382 Waihuan East Road, Panyu District, Guangzhou, Guangdong, 510006, China; School of Future Technology, South China University of Technology, 777 Xingye Avenue East, Panyu District, Guangzhou, Guangdong, 511442, China; Guangdong Engineering Research Center of Low-Carbon Synthetic Biotechnology, South China University of Technology, 382 Waihuan East Road, Panyu District, Guangzhou, Guangdong, 510006, China; School of Biology and Biological Engineering, South China University of Technology, 382 Waihuan East Road, Panyu District, Guangzhou, Guangdong, 510006, China; Guangdong Engineering Research Center of Low-Carbon Synthetic Biotechnology, South China University of Technology, 382 Waihuan East Road, Panyu District, Guangzhou, Guangdong, 510006, China; School of Biology and Biological Engineering, South China University of Technology, 382 Waihuan East Road, Panyu District, Guangzhou, Guangdong, 510006, China; School of Future Technology, South China University of Technology, 777 Xingye Avenue East, Panyu District, Guangzhou, Guangdong, 511442, China; Guangdong Engineering Research Center of Low-Carbon Synthetic Biotechnology, South China University of Technology, 382 Waihuan East Road, Panyu District, Guangzhou, Guangdong, 510006, China; Research Projects Department, Guangdong Artificial Intelligence and Digital Economy Laboratory (Guangzhou), 70 Anyue Road, Haizhu District, Guangzhou, Guangdong, 510335, China; Guangdong Engineering Research Center of Low-Carbon Synthetic Biotechnology, South China University of Technology, 382 Waihuan East Road, Panyu District, Guangzhou, Guangdong, 510006, China; School of Biology and Biological Engineering, South China University of Technology, 382 Waihuan East Road, Panyu District, Guangzhou, Guangdong, 510006, China

## Abstract

**Motivation:**

Recent advances in biological foundation models have enabled new opportunities for data-driven molecular engineering. However, designing programmable RNA devices remains challenging because of the vast, nonlinear sequence-structure-function landscape. Here, we present Ernie-Switcher, a deep learning framework built upon the RNA foundation model (RNAErnie), comprising three coupled components: (i) integration of RNAErnie-derived sequence-phenotype representations for realistic design generation and filtering, (ii) characterization of RNA landscape navigability as a theoretical basis for sequence optimization, and (iii) incorporation of biophysical constraints to maintain optimization trajectories within biologically plausible bounds.

**Results:**

We validated Ernie-Switcher using RNA toehold switches. Theoretically, the observed lower bound on threshold navigability (86.4%) of the RNA landscape supports the theoretical feasibility of RNA switch sequence optimization. Experimentally, the optimized toehold switches achieved ON/OFF expression ratios of up to 35-fold in *Escherichia coli*. Collectively, RNAErnie-guided sequence optimization provides a scalable strategy for engineering high-performance RNA devices. To facilitate broad adoption, we deployed Ernie-Switcher as a freely accessible web platform.

**Availability and implementation:**

The Python code and pre-trained models are available at https://github.com/zhutiblah/Ernie-Switcher and are archived on Zenodo at https://doi.org/10.5281/zenodo.21859277. The RNA toehold switch design platform is available at https://ernie-switcher.bioinformatics-syn.org/.

## 1 Introduction

Contemporary biotechnology is shifting from intuition-guided, trial-and-error optimization toward data-driven and increasingly automated molecular engineering (Faber and Whitehead 2019, Ferguson and Brown 2022, Shi *et al.* 2026). Among programmable devices, RNA is particularly attractive because it can be engineered to regulate gene expression, making it a versatile substrate for therapeutics (Crooke *et al.* 2018, Qian *et al.* 2022), diagnostics (Byron *et al.* 2016, Siddaway *et al.* 2025), and synthetic biology (Green *et al.* 2014, Dykstra *et al.* 2022, Freije and Arechavala-Gomeza 2025). However, engineering high-performance RNA devices remains challenging. RNA design spaces are vast, and the mapping from sequence to structure and function is highly nonlinear (Green *et al.* 2014). Even subtle sequence changes can reconfigure base-pairing patterns, destabilize functional conformations, or introduce unintended structures that compromise performance. As a result, the central challenge is not only to identify promising sequences, but also to determine whether feedback optimization can reliably move from typical starting points toward high-performing designs without becoming trapped in valleys of the performance landscape (Li *et al.* 2016).

Recent RNA foundation models suggest that generalizable information captured from large-scale sequence data could help address this challenge (Shen *et al.* 2024, Shulgina *et al.* 2024, Wang *et al.* 2024). For example, RhoFold + demonstrated that foundation model representations can support accurate RNA three-dimensional structure prediction from sequence (Shen *et al.* 2024). At the same time, RNAErnie leveraged motif-aware pretraining and fine-tuning to learn representations that generalize across diverse RNA modalities and tasks (Wang *et al.* 2024). Shulgina *et al.* (2024) further showed that the RNA foundation models could propose mutations that improve RNA function, highlighting their potential for sequence generation and functional optimization. Together, these studies indicate that foundation models capture informative regularities in RNA sequence and structure. However, improved representations alone do not guarantee successful optimization. Whether foundation model-guided design systems can reliably improve RNA function may also be influenced by the navigability of the RNA landscape (Greenbury *et al.* 2022).

From an evolutionary perspective, navigability describes the extent to which successive single-nucleotide mutations can move a sequence toward higher-performance phenotypes without traversing severe performance valleys (Greenbury *et al.* 2022). Mechanistically, navigability is rooted in genotype-phenotype (GP) maps. In nature, numerous distinct genotypes can encode the same phenotype. This redundancy creates connected neutral networks that enable performance-preserving exploration through neutral mutation. Such exploration can reposition sequences in genotype space until beneficial mutations become accessible (Reidys *et al.* 1997). In RNA engineering, equivalent connectivity would provide a mechanistic basis for feedback optimization, because sequence updates could remain within functional regions while progressively accessing improved designs. However, navigability in RNA switch landscapes has rarely been explicitly quantified during design.

To address this gap, we employed RNA toehold switches as a model system. RNA toehold switches are widely used cis-regulatory elements (To *et al.* 2018) that undergo trigger-dependent conformational switching to modulate gene output [e.g. in response to a cognate trigger RNA (Wang and Simmel 2022)]. A pioneering study reported a library exceeding 100 000 toehold switch variants, providing a substantial data resource for the development and validation of data-driven engineering design approaches (Angenent-Mari *et al.* 2020). Drawing upon this resource, subsequent studies integrated sequence generation and performance prediction into feedback optimization pipelines. These data-driven frameworks improved toehold switch performance, achieving ON/OFF ratios up to 78-fold and outperformed NUPACK (Zadeh *et al.* 2011, Riley *et al.* 2025, Robson and Green 2026). Despite these advances, key gaps remain. Prior pipelines have rarely quantified navigability in RNA design landscapes, have yet to incorporate RNA foundation models within the optimization loop, and typically optimize computational objectives without explicit biophysical constraints. Consequently, optimization may favor sequences with strong *in silico* scores but limited functional viability.

Here, we present Ernie-Switcher, a deep learning framework that leverages RNAErnie to establish an interpretable design system for programmable RNA devices. The quantification of RNA landscape navigability provides a theoretical basis for the feasibility of sequence optimization. Against this landscape-level theoretical background, Ernie-Switcher uses RNAErnie guidance to improve candidate generation, candidate prioritization, and latent-space optimization. It further incorporates biophysical constraints, including self-folding minimum free energy (MFE) and GC content, to guide the latent-space search toward high-performing and biophysically plausible sequence regions. Benchmarked on RNA toehold switches, RNAErnie guidance improved candidate quality and optimization efficiency, and the resulting lead variants achieved ON/OFF ratios of up to 35-fold in *Escherichia coli*. To enhance accessibility, Ernie-Switcher is provided as a web-based platform. More broadly, these results position Ernie-Switcher as an example of how RNA engineering can move beyond intuition-guided trial-and-error toward data-driven molecular design.

## 2 Materials and methods

### 2.1 Dataset and multi-feature representation

The RNA switch datasets utilized in this study were derived from previously published works by Angenent-Mari *et al.* (2020) and Riley *et al.* (2025). Following a rigorous curation process that entailed the removal of null values and extraneous information, a final corpus of 45 610 valid RNA switch entries (see File 1, available as supplementary data at *Bioinformatics* online, for more details) was retained.

To comprehensively capture the physicochemical attributes and semantic context of these sequences, we constructed a multi-feature representation framework that integrates four distinct feature channels. The framework first explicitly encodes the sequence by mapping nucleotides to discrete indices (i.e. A,U,G,C are mapped to integers 0–3, respectively). Next, a dimer feature is constructed to capture neighbor-dependent interactions, using an analogous integer encoding for all 16 dinucleotides (e.g. AA↦4, AU↦5, etc) (Lei *et al.* 2025). Complementing these is a structure channel, represented by a hydrogen-bond potential matrix $S\in Z^{N\times N}$ (*N* denotes the switch sequence length), where each entry $S_{ij}\in \{0,2,3\}$ encodes the base-pairing potential between positions *i* and *j*: $S_{ij}=3$ for G–C/C–G pairs, $S_{ij}=2$ for A–U/U–A and wobble G–U/U–G pairs, and $S_{ij}=0$ otherwise (Riley *et al.* 2025). Finally, we employed embeddings from a pre-trained RNAErnie (Wang *et al.* 2024) model as semantic features. RNAErnie is an ERNIE-style masked language model tailored for biological sequence modeling, and adopts a base-scale Transformer architecture consisting of 12 encoder layers, 12 attention heads, and a hidden dimension of 768. Specifically, we extracted the hidden states from the final encoder layer, yielding a tensor of size N×45×768, where *N* denotes the sequence length. These representations were kept frozen during downstream training and used as biologically grounded static input features.

### 2.2 Hybrid objective function for switch performance prediction

To balance prediction accuracy with ranking capability, we designed a hybrid loss function $L_{\mathrm{pred}}$, which synergizes Mean Squared Error (MSE) to minimize absolute deviation and a Pearson Correlation Coefficient (PCC) loss to maximize linear correlation trends:


(1) $$L_{\mathrm{pred}}=L_{\mathrm{MSE}}(Y_{\mathrm{true}},Y_{\mathrm{pred}})-\rho_{\mathrm{PCC}}(Y_{\mathrm{true}},Y_{\mathrm{pred}}).$$

Here, $Y_{\text{pred}}$ denotes the model-predicted output and $Y_{\text{true}}$ denotes the corresponding ground-truth value (experimentally measured).

### 2.3 RNAErnie-guided diffusion model for RNA switch design

The Gaussian diffusion model is trained with a weighted combination of a standard diffusion noise-prediction loss and a LLM-guided alignment loss:


(2) $$L_{\text{total}}=L_{\text{MSE}}+wL_{\text{align}},$$

where *w* is a scalar weight (set to 0.05 in our experiments). The diffusion loss $L_{\text{MSE}}$ is defined as the mean-squared error between the true Gaussian noise and the U-Net prediction. To inject high-level sequence semantics, we introduce an LLM-guided alignment loss ($L_{\text{align}}$) that matches a global representation of the U-Net bottleneck to the pre-trained RNAErnie embedding. Specifically, we obtain a diffusion representation $z_{\text{diff}}\in R^{1024}$ by global average pooling over the U-Net bottleneck feature map, and compute a target semantic embedding $e_{\text{LM}}\in R^{768}$ by averaging RNAErnie hidden states. A learnable projection $W_{\text{proj}}:R^{1024}\to R^{768}$ maps $z_{\text{diff}}$ into the RNAErnie space, yielding $z_{\text{aligned}}=W_{\text{proj}}(z_{\text{diff}})$. The alignment loss is then


(3) $$L_{\text{align}}={\left\|z_{\text{aligned}}-e_{\text{LM}}\right\|}_{2}^{2}.$$

### 2.4 Evolutionary closed-loop optimization in switch sequence space

To precisely tailor switches with high dynamic ranges, we established a closed-loop iterative optimization framework, which comprises three coupled components:

1. Generator ($G_{\theta }$): a frozen LLM-guided diffusion model that maps continuous latent variables in Z to discrete RNA switch sequences in S.
2. Evaluator ($F_{\phi }$): a pre-trained translation level prediction model that estimates ON- and OFF-state expression.
3. Optimizer: CMA-ES navigates the latent space to identify high-performing designs.

Using these three components, we formulate the search as a maximization problem in the latent space, seeking z∈Z that yields high-performing switches. The optimization proceeds iteratively; at each generation *g*, the procedure consists of five stages:

1. Sampling: a population of latent vectors is sampled from a multivariate normal distribution parameterized by CMA-ES (Nikolaus 2016),
  (4) $$z∼N(m,\sigma^{2}C),$$
  where N(·) denotes a multivariate normal distribution, m is the current mean (i.e. the center) of the sampling distribution, σ is a global step size controlling how widely new candidates are explored, and C is the covariance matrix that captures correlations among latent variables.
2. Switch generating: latent vectors are mapped to candidate RNA switch sequences via the diffusion model,
  (5) $$s=G_{\theta }(z),\;s\in S.$$
3. Functional evaluation: each candidate sequence *s* is scored by the pre-trained predictor,
  (6) $$S_{\text{func}}(s)=P_{\text{on}}(s)-P_{\text{off}}(s),$$
  where $P_{\text{on}}(s)$ and $P_{\text{off}}(s)$ denote the predicted ON- and OFF-state translation level, respectively.
4. Biophysical constraint: to encourage physically plausible designs, soft biophysical constraints were imposed on ON-state MFE, OFF-state MFE, and ON-state GC content. For each candidate switch, the OFF-state transcript was assembled as “Loop1—Recognition—Loop2—Stem1—AUG—Stem2—Linker—Post-linker” (Angenent-Mari *et al.* 2020), whereas the ON-state transcript was derived by inserting the cognate trigger immediately upstream of Loop1, yielding “Trigger—Loop1—Recognition—Loop2—Stem1—AUG—Stem2—Linker—Post-linker”. Loop1 is the constant unstructured linker used in the fused ON-state library architecture to connect the trigger RNA to the 5′ end of the switch mRNA (Angenent-Mari *et al.* 2020). MFE was computed using ViennaRNA (Lorenz *et al.* 2011), and GC content was computed on the ON-state transcript. Constraint ranges were determined empirically from the training data by identifying high-performance plateau regions. For each biophysical variable, sequences were grouped into equal-width bins and the median ON–OFF per bin was computed; the plateau was defined as the contiguous set of bins whose median ON–OFF was at least 50% of the peak median (threshold fraction = 0.5). The resulting target ranges were set to
  (7) $$\begin{array}{c} {\text{MFE}}_{\text{on}}\in [-82.0,-56.0]\;\text{kcal}{\text{mol}}^{-1},\\ {\text{MFE}}_{\text{off}}\in [-46.0,-28.0]\;\text{kcal}{\text{mol}}^{-1},\\ {\text{GC}}_{\text{on}}\in [0.360,0.520]. \end{array}$$
  Deviations from each interval were quantified by a bounded violation function. For a scalar property *x* with target interval [l,h], we first defined:
  (8) $$d(x)=\{\begin{array}{cc} 0, & l\leq \;x\leq h,\\ \text{min}\left(\left|x-l|,\;|x-h\right|\right), & \text{otherwise}, \end{array}$$
  where d(x) measures the distance of *x* to the nearest boundary of the allowed interval (and is zero if *x* lies within the interval). We then normalize this deviation to obtain a dimensionless violation score v(x):
  (9) $$v(x)=\frac{d(x)}{d(x)+(h-l)}.$$
  For each candidate switch sequence, the biophysical penalty $P_{\text{bio}}$ is computed as:
  (10) $$P_{\text{bio}}(x)=\alpha \sum_{k}v_{k}(x),\;\alpha =0.1.$$
  where constraint $k\in \{{\text{MFE}}_{\text{on}},{\text{MFE}}_{\text{off}},{\text{GC}}_{\text{on}}\}$, and α is a penalty weight that controls the strength of the biophysical regularization.
5. Feedback & adaptation: the final score integrates functional performance and biophysical plausibility,
  (11) $$S(s,x)=S_{\text{func}}(s)-P_{\text{bio}}(x),$$
  and CMA-ES updates its distribution parameters (m,σ,C) using the S(s,x) to gradually drift the sampling result toward high-performing, constraint-consistent regions.

### 2.5 Baseline design methods

We selected two previously reported RNA design frameworks, GARDN and GARDN-SANDSTORM, as baseline methods for comparison (Robson and Green 2026). GARDN is a generative adversarial RNA design network trained under the Wasserstein generative adversarial network with gradient penalty (WGAN-GP) framework. Its generator maps random latent variables to RNA sequences, whereas the discriminator encourages the generated candidates to reproduce the sequence and structural characteristics of the experimentally characterized training set. SANDSTORM is a convolutional neural network predictor that processes RNA sequence and secondary-structure representations through parallel feature-extraction channels and predicts functional properties such as ON- and OFF-state expression. In the combined GARDN-SANDSTORM framework, the pretrained generator and predictor are frozen, and the predictor-derived objective is backpropagated to the latent input of GARDN within an iterative optimization loop, thereby steering generated sequences toward improved predicted performance without updating the parameters of either model. For experimental comparison, the GARDN and GARDN-SANDSTORM sequences were directly obtained from the publicly reported designs in the original study and synthesized and evaluated in our experimental system.

### 2.6 Structural agreement metric

To assess whether generated toehold switches preserve secondary-structure patterns observed in the reported set, we defined a structural agreement metric. Each dot–bracket structure was converted into a length-*N* integer vector $q\in {\left\{0,1,2\right\}}^{N}$ by mapping “.,” “(,” and “)” to 0, 1 and 2, respectively. For a native structure $q_{\text{ref}}$ and a generated structure $q_{\text{gen}}$, we computed their structural agreement as the fraction of nucleotide positions with the same structural state:


(12) $$\text{Sim}(q_{\text{ref}},q_{\text{gen}})=\frac{1}{N}\sum_{k=1}^{N}I\left(q_{\text{ref}}^{\left(k\right)}=q_{\text{gen}}^{\left(k\right)}\right),$$

where I(·) returns 1 when the two positions share the same structural state (and 0 otherwise). We then summarized dataset-level structural agreement using the average maximum similarity (AMS): for each native structure, we find it’s most similar generated structure and average this best-match score over all native structures,


(13) $$\text{AMS}=\frac{1}{\left|Q_{\text{ref}}\right|}\sum_{i=1}^{\left|Q_{\text{ref}}\right|}{\text{max}}_{q_{\text{gen}}\in Q_{\text{gen}}}\text{Sim}\left(q_{\text{ref}}^{\left(i\right)},q_{\text{gen}}\right),$$

where $Q_{\text{ref}}$ and $Q_{\text{gen}}$ denote the native and generated structure sets, respectively.

### 2.7 Weighted on/off metric

To assess the relationship between nucleotide composition in the last three positions of the recognition region and RNA toehold switch performance, we performed a weighted ON/OFF analysis. For each toehold switch *i*, let $y_{i}$ denote the experimentally measured ON/OFF value, $n_{i}^{\left(U\right)}$ the number of U in the last three nucleotides of the recognition subsequence, and $n_{i}^{\left(G\right)}$ the number of G in the same positions. For *N* toehold switch sequences, the weighted ON/OFF metric was calculated as


(14) $$W^{\left(U\right)}=\sum_{i=1}^{N}n_{i}^{\left(U\right)}y_{i},$$

(15) $$W^{\left(G\right)}=\sum_{i=1}^{N}n_{i}^{\left(G\right)}y_{i}.$$

### 2.8 Training and evaluation strategy

To ensure robust generalization, we adopted a five-fold cross-validation protocol, reporting final metrics as the ensemble average across all folds. Furthermore, to mitigate overfitting, training was governed by an adaptive early stopping mechanism. The process was terminated if validation loss did not improve within a patience of 50 epochs, retaining the model parameters associated with the optimal validation performance.

### 2.9 Threshold navigability and neutral mutations

#### 2.9.1 Genotype, phenotype, and fitness

We treat each RNA sequence as a genotype, denoted by g∈G. Its predicted secondary structure as a phenotype p∈P. Mapping each genotype to a single predicted structure constitutes a coarse-grained modeling simplification for genotype-phenotype (GP) map analysis. The GP relationship is represented:


(16) M:G→P, p=M(g).

We define fitness at the phenotype level. Each phenotype *p* is associated with a fitness value $F_{p}$. Accordingly, the fitness of a genotype g is induced by its phenotype and written as:


(17) $$F(g)=F_{M(g)}.$$

This choice is motivated by the fact that, in RNA systems, biological function is closely associated with the assembled RNA structural phenotype (Greenbury *et al.* 2022). Each distinct phenotype *p* is assigned a fitness value $F_{p}$ sampled independently from a Uniform(0,1) distribution. This randomized phenotype-level assignment isolates the contribution of the underlying genotype–phenotype map and is used to probe its task-agnostic navigability.

#### 2.9.2 Neutral mutations

We consider the single-nucleotide mutation graph on G, where an edge $g\to g^{\prime }$ corresponds to one base mutation and induces a phenotype transition $p=M(g)\to p^{\prime }=M(g^{\prime })$. Within this framework, we define a neutral mutation as a mutation that leaves the phenotype unchanged,


(18) $$M(g^{\prime })=M(g).$$

Such mutations also preserve fitness,


(19) $$F_{M(g^{\prime })}=F_{M(g)}.$$

Biologically, such neutral mutations enable broad exploration of genotype space without immediate fitness loss, thereby potentially allowing fitness to increase monotonically along evolutionary trajectories.

#### 2.9.3 Threshold navigability

Under the conventional notion of navigability, a path from a designated start genotype to a designated target genotype is navigable if fitness is non-decreasing along the path. Formally, a mutational path $\left(g_{0},g_{1},\ldots ,g_{L}\right)$ is monotonic if


(20) $$F_{M(g_{i+1})}\geq F_{M(g_{i})}\;\forall i\in \{0,1,\ldots ,L-1\},$$

where *L* denotes the path length. Biologically, such paths allow adaptation to proceed without traversing fitness valleys.

In our setting, the designed RNA sequences have length 45nt (recognition + stem1 + stem2), substantially exceeding the 20–30nt typically considered in prior work (Greenbury *et al.* 2022), thereby expanding the genotype search space exponentially. As a consequence, requiring navigability strictly between a designated start sequence $g_{A}$ and a target sequence $g_{B}$ becomes computationally impractical. We therefore introduce threshold navigability. Importantly, unless otherwise specified, all references to navigability in this study are with respect to threshold navigability.

For a user-specified fitness threshold τ, we define the high-fitness phenotype set as


(21) $$H_{\tau }=\{p\in P:F_{p}\geq \tau \},$$

where, in this study, τ was chosen as the 95th percentile of the fitness distribution, i.e. the threshold corresponding to the top 5% of fitness values. Given an initial genotype $g_{0}$, a path $\left(g_{0},g_{1},\ldots ,g_{L}\right)$ is said to be threshold-navigable at level τ if consecutive genotypes differ by one nucleotide, fitness is non-decreasing along the path,


(22) $$F_{M(g_{i+1})}\geq F_{M(g_{i})}\;\forall i\in \{0,1,\ldots ,L-1\},$$

and the terminal genotype maps to a phenotype in the high-fitness set:


(23) $$M(g_{L})\in H_{\tau }\;\Leftrightarrow \;F_{M(g_{L})}\geq \tau .$$

We refer to such a path as a threshold-navigable path.

#### 2.9.4 Navigable, aborted, and unnavigable

Threshold-navigable paths were identified using a heuristic DFS-restart search in genotype space (Greenbury *et al.* 2022). From each source RNA sequence, the search considered only single-nucleotide mutant neighbors and terminated upon reaching any phenotype in $H_{\tau }$. Each trial was classified as *navigable*, *aborted* or *unnavigable*. Trials were classified as *navigable* when a threshold-navigable path was found. Trials were classified as *aborted* when the computational budget was exhausted before a threshold-navigable path was identified, with a budget of $10^{8}$ visited genotypes per trial. A trial could be classified as *unnavigable* only under exhaustive exploration of all mutational trajectories. As this is computationally intractable for the RNA sequence space examined here ($4^{45}$ possible sequences), no trial was formally classified as unnavigable. Because aborted trials may still contain threshold-navigable paths that were not identified within the computational budget, the observed navigable fractions should be interpreted as heuristic- and budget-dependent lower bounds on the true navigable fractions among the sampled starting genotypes.

#### 2.9.5 Sensitivity analysis of threshold navigability

To assess the robustness of the observed navigable fraction, we evaluated nine combinations of fitness threshold (85th, 90th, and 95th percentiles) and computational budget ($10^{6}$, $10^{7}$, and $10^{8}$ visited genotypes per trial). The resulting fractions were compared across conditions to quantify their dependence on threshold stringency and search budget (Table 1, available as supplementary data at *Bioinformatics* online). Because the analysis was based on 2260 sampled starting genotypes, representing only a minute fraction of the full $4^{45}$ sequence space, the resulting navigable fractions should be regarded as reference estimates under the current sampling and computational settings rather than as direct measures of global navigability. These computations required approximately seven days on a server equipped with two Intel Xeon Platinum 8380 CPUs, providing 80 physical cores and 160 logical threads in total.

### 2.10 Validation of synthetic switch sequences in *E. coli*

#### 2.10.1 Plasmid construction

Toehold switch expression plasmids were constructed by inserting double-stranded DNA fragments encoding the RNA switches into PCR-amplified pSEVA221-derived backbones using Gibson assembly (2× EzmaxR Ultra Universal CloneMix, TOLOBIO, 24 317 China). The RNAs were transcribed under the control of a T7 promoter in *E. coli* BL21(DE3), with T7 RNA polymerase expression induced by IPTG. The switch-controlled reporter sequence was derived from enhanced green fluorescent protein (EGFP) in pcDNA3-EGFP (Addgene plasmid #13031), and the EGFP reporter did not contain a degradation tag. All PCR amplifications were performed with KOD One PCR Master Mix -Blue- (TOYOBO, KMM-201, Japan). Plasmids were maintained in *E. coli* BL21(DE3) (TOLOBIO, CC96107, China) and verified by Sanger sequencing. The sequences of all RNA switches used in this study are provided in File 2, available as supplementary data at *Bioinformatics* online.

#### 2.10.2 Fluorescence assay experiments

*E. coli* BL21(DE3) clones harboring either switch–trigger plasmids or non-trigger plasmids were inoculated into 5 ml LB medium (10 g/l NaCl, 10 g/l tryptone, and 5 g/l yeast extract) supplemented with 50 mg/l kanamycin and cultured overnight at 37°C with shaking at 220 rpm. After 16 h, 1% (v/v) of the overnight culture was inoculated into 1.5 ml fresh LB medium containing 50 mg/l kanamycin in a 24-well plate and incubated at 37°C with shaking at 160 rpm. After 2 h, when the OD_600_ reached approximately 0.2, isopropyl β-D-1-thiogalactopyranoside (IPTG) was added to a final concentration of 0.25 mM. Following 9 h of IPTG induction, fluorescence intensity was measured using a BioTek Synergy H1 microplate reader (Agilent, USA). For both ON- and OFF-state cells, mean fluorescence values were calculated from three technical replicates, and error bars represent the SD derived from three independent biological replicates. All chemicals used in the experiments were purchased from China National Medicines Corporation Ltd.

## 3 Result

### 3.1 The Ernie-Switcher framework for RNA toehold switch design

Our design objective is to construct a controllable toehold switch that regulates translation through a trigger-dependent conformational rearrangement. In the OFF state, a stem-loop sequesters the ribosome binding site (RBS) and start codon (AUG), thereby precluding translation initiation. Upon cognate trigger binding, the switch refolds to expose the RBS/AUG, facilitating translation of the downstream coding sequence (Fig. 1A). The Ernie-Switcher workflow encompasses four integrated modules: candidate generation, performance prediction, sequence optimization, and experimental validation (Fig. 1B–E). Initially, RNAErnie-derived representations guide the diffusion model to generate candidate sequences that faithfully recapitulate the sequence and structural motifs associated with functional RNA toehold switches (Fig. 1B). In parallel, a multi-feature predictor—integrating four complementary channels (sequence, dimer, structure, and RNAErnie features) via a cross-attention mechanism—yields quantitative estimates of ON-state expression and OFF-state leakage (Fig. 1C). Subsequently, guided by the predictor and governed by biophysical constraints, candidate sequences are optimized in latent space along trajectories that progressively improve predicted performance while reducing the occurrence and depth of performance valleys (Fig. 1D). Lastly, the optimized synthetic RNA switches are assembled into a plasmid library, tested in *E. coli*, and quantified by fluorescence measurements to validate their *in vivo* regulatory efficacy (Fig. 1E).

**Figure 1:**
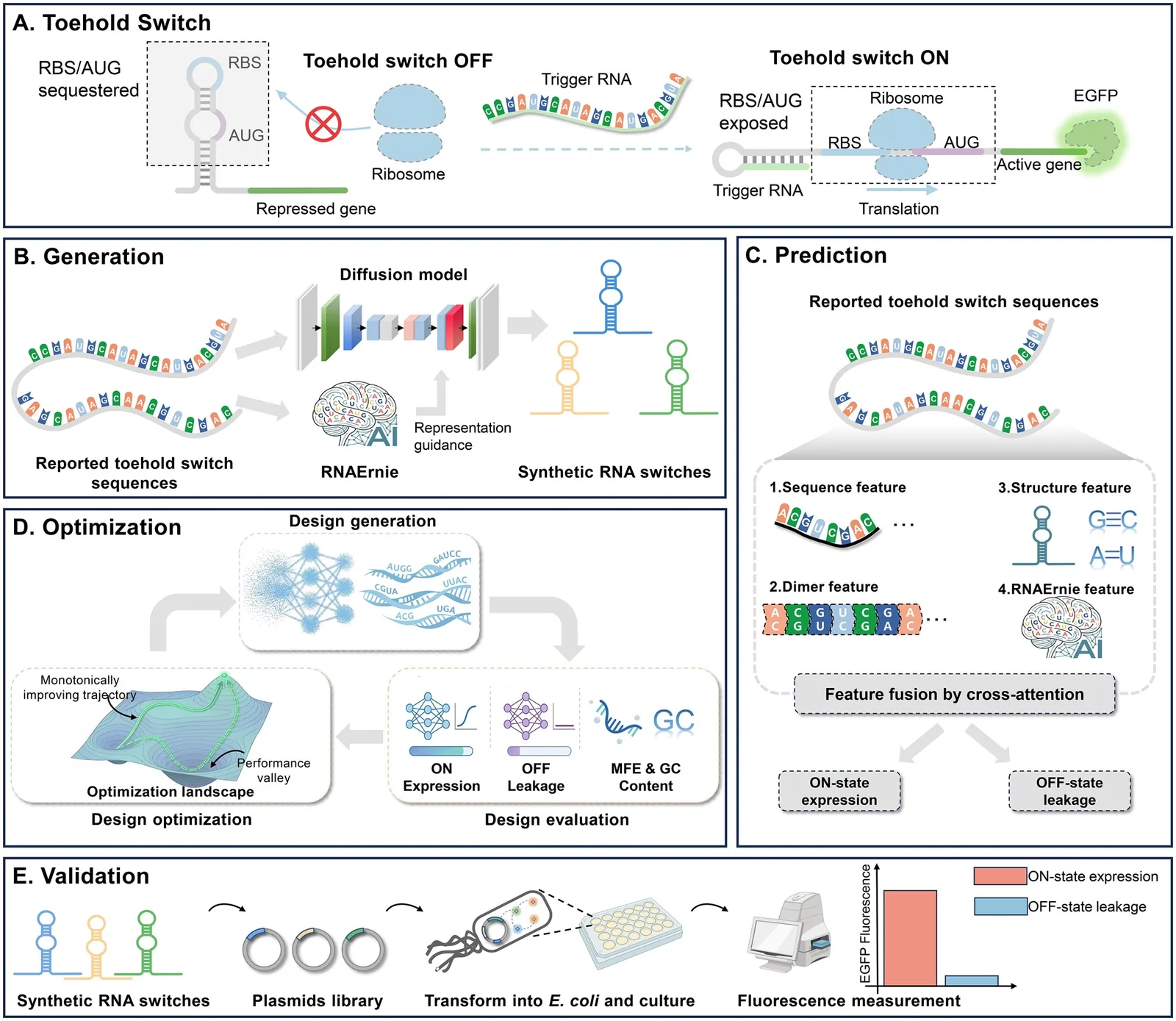
Framework for foundation model-driven RNA toehold switch design. (A) Mechanism of toehold switch regulation. In the OFF state, the RBS and AUG are sequestered and inaccessible, thereby preventing translation of the downstream gene. Upon binding of a cognate trigger RNA, the switch rearranges into the ON state in which the RBS/AUG becomes accessible, enabling translation of the downstream gene. (B) Generation module. An RNAErnie-guided diffusion model is used for RNA toehold switch sequence generation. (C) Prediction module. A multi-feature predictor integrates four complementary feature channels, including sequence features, dimer features, structure features, and RNAErnie embeddings. These features are fused via cross-attention to predict ON-state expression and OFF-state leakage. (D) Optimization module. Candidate sequences generated by the RNAErnie-guided diffusion model are evaluated using trained predictors for ON-state expression and OFF-state leakage together with biophysical constraints, including MFE and GC content. These evaluations guide closed-loop optimization toward high-performing designs, with an increased proportion of trajectories showing non-decreasing performance and reduced performance valleys. (E) Experimental validation. Selected synthetic RNA switches are cloned into a plasmid library and expressed in *E. coli*. Their regulatory performance is quantified by fluorescence measuremes of ON-state expression and OFF-state leakage.

### 3.2 RNAErnie-guided diffusion generates functional switch sequences

Having established the design framework, we next assessed the quality of sequences produced by the RNAErnie-guided diffusion model (Fig. 2). The model was first trained under a moderate guidance weight (*w *= 0.05), and both training and validation losses rapidly decreased and converged in tandem (Fig. 2A), indicating stable learning without overfitting. The guidance weight (*w*) was then varied to modulate the influence of the RNAErnie priors. Moderate guidance (*w *= 0.03 or 0.05) preserved strong k-mer frequency correlations between synthetic and reported switches, whereas an excessive weight (*w *= 0.1) led to declining correlations and stronger fluctuations (Fig. 2B). This observation suggested that calibrated RNAErnie guidance facilitated the retention of reported-like local sequence statistics while ensuring training stability. At the structural level, compared with the unguided DDPM baseline, moderate guidance (*w *= 0.03 and *w *= 0.05) substantially accelerated improvements in structural consistency. By 50 epochs, structural agreement reached 0.965, exceeding the baseline DDPM at the same epoch (0.918) and rivaling the baseline’s asymptotic performance achieved after 500 epochs (Fig. 2C). Given that RNA switch regulation is intrinsically linked to specific secondary structure conformations and long-range pairing patterns, this rapid enhancement in structural consistency provided a favorable starting point (RNAErnie-guided diffusion and Diffusion in Fig. 5B) for downstream functional screening by enriching for candidates with reported-like folding properties.

**Figure 2:**
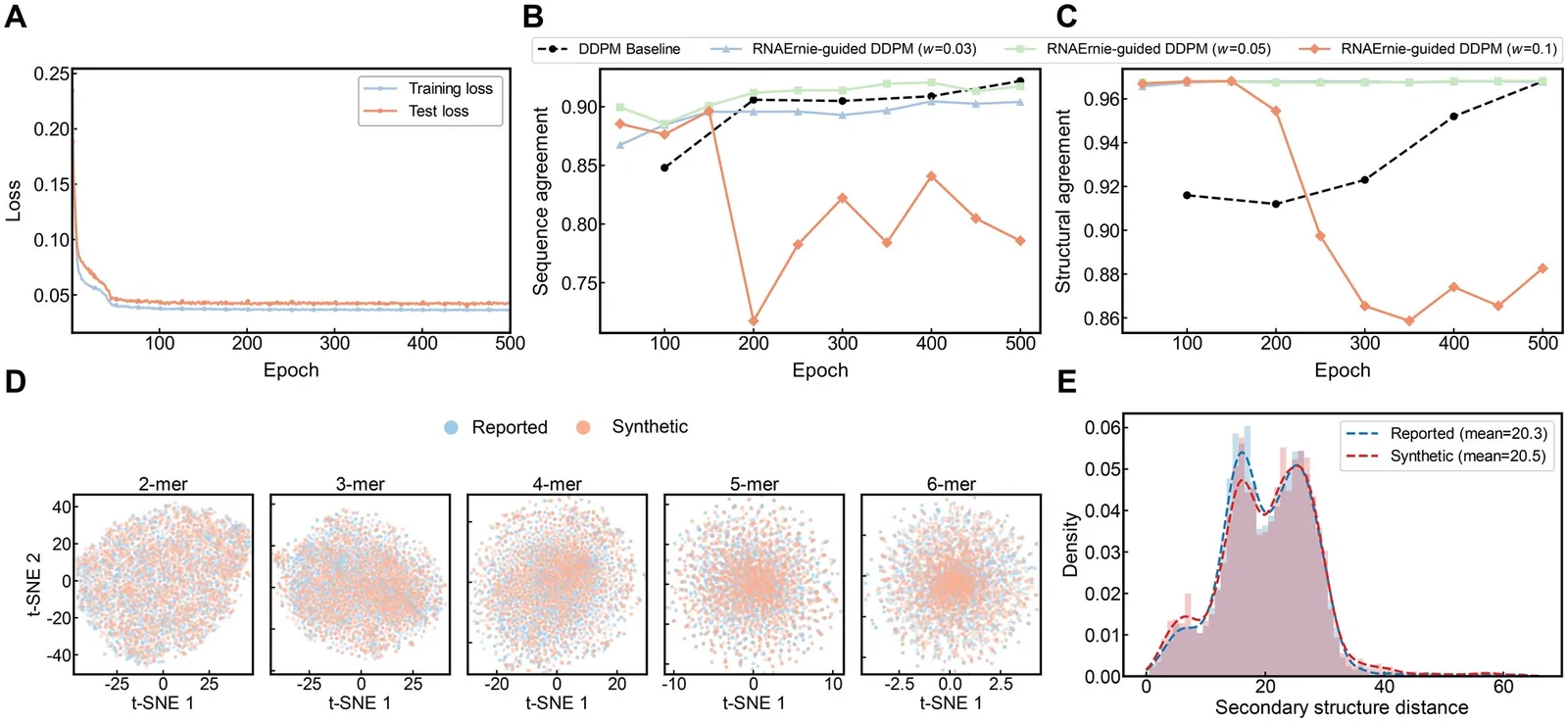
RNAErnie guidance improves both sequence and structural fidelity while maintaining diversity. (A) Training and test loss of the RNAErnie-guided diffusion model under a fixed guidance weight (*w *= 0.05), showing stable convergence during training. Here, *w* is a weighting factor that determines how much RNAErnie prior information is incorporated [see Eq. (2)]. (B, C) Sequence agreement (6-mer frequency correlations quantified by PCC) and structural agreement [quantified by AMS; Eq. (13)] between 2048 synthetic RNA toehold switches and the held-out test set under different values of *w*. A moderate value of *w* (*w *= 0.05) preserves biologically plausible sequence composition and structural consistency with the reported test set, indicating that balanced prior incorporation is beneficial. (D) t-SNE visualization of 2-mer to 6-mer composition features for the held-out test set (blue) and 2048 synthetic switches (orange). The substantial overlap between the two distributions indicates that the synthetic sequences recapitulate the compositional characteristics of reported switches, whereas their broad dispersion suggests maintained sequence diversity. (E) Distributions of secondary structure distances within the reported and synthetic RNA toehold switch ensembles. The close agreement between the two distributions indicates that the synthetic switches preserve the structural organization of reported switches. Secondary structure distance was defined as the number of mismatched base pairs between two dot-bracket structures.

To further characterize sequence similarity and diversity, we projected k-mer composition features (*k* = 2–6) for both reported and generated switches via t-SNE (Fig. 2D). Across the k-mer feature space, generated sequences exhibited significant overlap with the reported test set. Notably, the generated samples occupied a dispersed distribution rather than concentrated clusters, suggesting that reported-like statistics were achieved without severe mode collapse. At the structural level, we compared the distributions of secondary-structure distances within the reported and generated datasets. The two distributions were highly congruent in both morphology and central tendency (Fig. 2E), reflecting comparable coverage of structural variability. Taken together, under the calibrated guidance weight, the RNAErnie-guided diffusion model robustly captured sequence-structure features of RNA toehold switches, yielding synthetic variants that recapitulated the sequence statistics and structural diversity of their reported counterparts.

### 3.3 Multi-feature modeling predicts switch performance and reveals sequence determinants

Accurate pre-synthesis prediction of ON-state expression and OFF-state leakage of RNA toehold switches could significantly improve screening efficiency and reduce experimental costs. Intuitively, coarse biophysical descriptors, such as GC content and MFE, were expected to govern switching behavior. However, these features showed marginal associations with switching performance, indicating that the patterns of regulatory dynamics were not captured by a simplistic mapping of hand-crafted variables (Fig. 1, available as supplementary data at *Bioinformatics* online). By contrast, the deep-learning model integrated sequence and structure signals to capture higher-order determinants of switch performance. Accordingly, we developed a sequence-performance prediction framework that integrated multi-feature information (including sequence features, dimer compositions, secondary structure representations, and RNAErnie embeddings) via a cross-attention mechanism to enable high-fidelity performance prediction (Fig. 1C).

On a held-out test set, Ernie-Switcher demonstrated superior predictive accuracy for both ON-state expression and OFF-state leakage, consistently outperforming established baselines (Fig. 3A and D). For the ON state, the Ernie-Switcher achieved a MSE of 0.034 and a Spearman correlation of 0.813 (Fig. 3A and B); for the OFF state it reached MSE = 0.023 and Spearman = 0.642 (Fig. 3C and D). This lower rank correlation for OFF may be partially attributable to label imbalance, as OFF measurements were heavily concentrated near zero with a compressed dynamic range (11.8% of OFF-state leakage = 0 in Fig. 2, available as supplementary data at *Bioinformatics* online). Notably, these metrics exceeded those of baseline models [e.g. SANDSTORM (Riley *et al.* 2025)] on the identical datasets. This improvement likely reflected synergistic fusion of multiple features. Secondary structure and dimer information constrained local accessibility and base-pairing energetics, while RNAErnie embeddings provided rich evolutionary and contextual information. The cross-attention module aligned these heterogeneous inputs, enabling the model to discern the coupled influence of local sequence context and global conformational stability on phenotypic outcomes.

**Figure 3:**
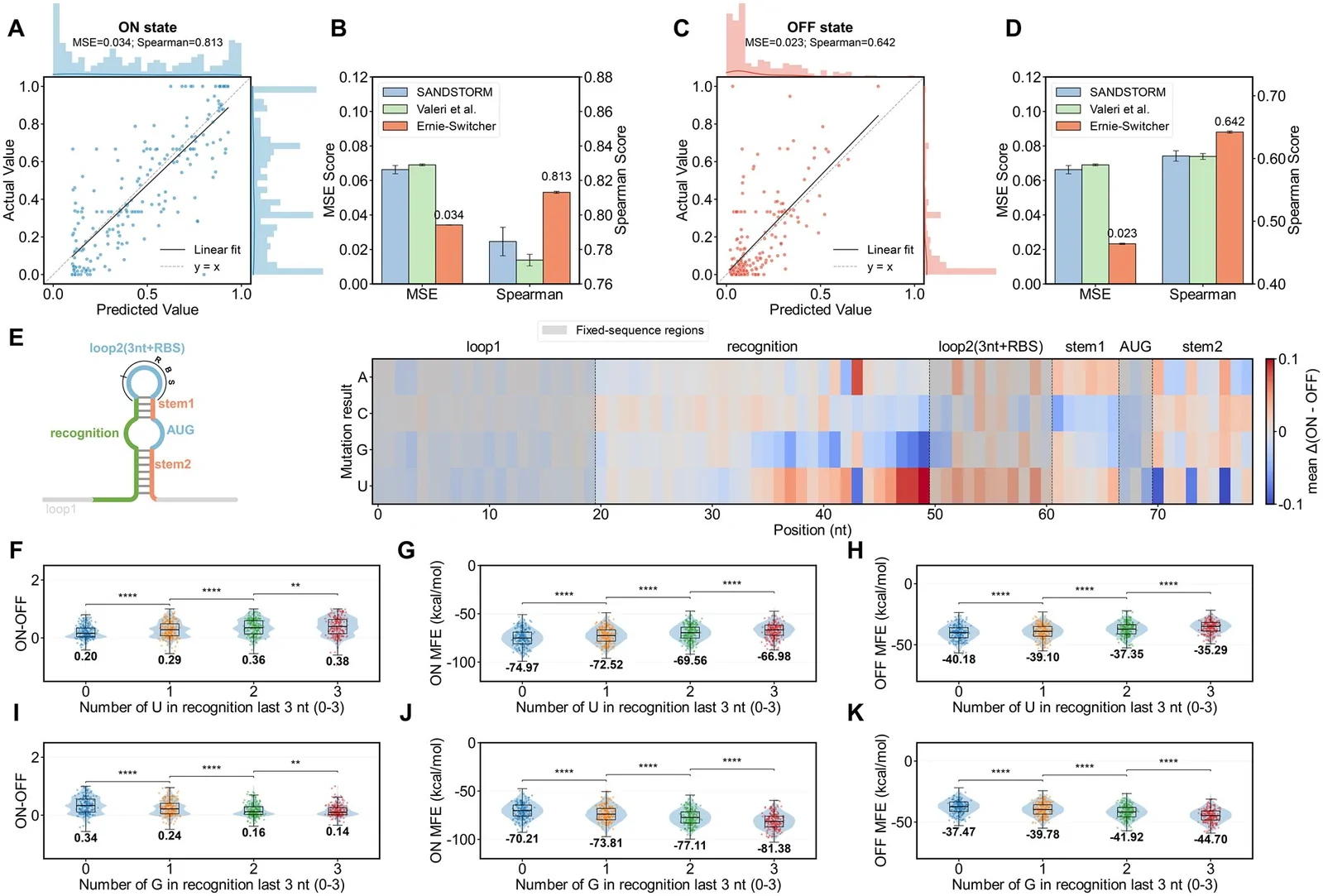
Multi-feature fusion improves performance prediction and reveals sequence features associated with toehold switch performance. (A), (C) Predicted values show strong agreement with experimental measurements for both ON-state expression (A) and OFF-state leakage (C). For visualization, 200 test samples were randomly selected for display, while all performance metrics were calculated on the full test set. Solid black lines represent linear fits of predicted versus observed values, and dashed lines denote the identity reference line (*y* =* x*), with marginal histograms showing the distributions of the displayed predicted and experimental values. (B), (D) Ernie-Switcher outperforms prior methods [SANDSTORM (Riley *et al.* 2025), Valeri *et al.* (2020)] in predicting both ON-state expression (B) and OFF-state leakage (D), as reflected by consistently higher Spearman correlation coefficients and lower MSE. Bar heights represent the mean performance across five cross-validation folds, and error bars indicate the SD. (E) Mean single-nucleotide saturation mutagenesis effect maps across the toehold switch sequence. Heatmaps report the mean predicted ON–OFF (ON-state expression—OFF-state leakage) change for each substitution (rows: A, C, G, U) at each position. Dashed vertical lines denote annotated regions (loop1, recognition, loop2, stem1, AUG, stem2). Systematic mutagenesis identifies the 3-nt window immediately upstream of the loop2 as the critical determinant of switch performance. Gray shading marks dataset-fixed regions, where predicted mutational effects may lack biological significance. (F-K) Distributions of ON–OFF, ON-state MFE, and OFF-state MFE across subsets stratified by nucleotide composition within a 3-nt hotspot window (identified in Panel E). U enrichment is associated with increased ON–OFF values and less negative ON-state and OFF-state MFE (F–H), whereas G enrichment shows the opposite trend (I–K), indicating that nucleotide composition within this hotspot window modulates switch performance through changes in local folding energetics. Dots represent individual sequences; boxplots summarize distributions; asterisks indicate statistical significance based on the two-sided Mann-Whitney *U* test (* P<0.05; ** $P<10^{-2}$; *** $P<10^{-3}$; **** $P<10^{-4}$).

We next combined the predictive model with *in silico* saturation single-nucleotide mutagenesis to probe nucleotide-level determinants of switch performance. Perturbations within the inhibitory hairpin—including the recognition, stem1 and stem2—exerted more pronounced effects on performance than mutations elsewhere (Figs 3E and Fig. 3, available as supplementary data at *Bioinformatics* online). Specifically, the mutational effect pattern within the Stem2 region exhibited an apparent 3-nt periodicity, suggesting a potential contribution of codon identity. Further codon-level analysis showed that the presence of stop codons (TAG, TAA, and TGA) at these positions was consistently associated with markedly reduced switch performance (Fig. 4, available as supplementary data at *Bioinformatics* online). This effect is consistent with premature termination of downstream reporter translation, which diminishes the distinction between the ON and OFF states and thereby compromises effective switching. Notably, the highest sensitivity was localized near the loop2 (3 nt + RBS), implicating the RBS-proximal structural context as a critical constraint on switch output. Specifically, within the 3-nt window immediately upstream of the loop2, U enrichment was associated with improved switch performance, whereas G enrichment exhibited a deleterious effect (Fig. 3E). Biophysically, enrichment of U and depletion of G were associated with increased MFE in the OFF state (Fig. 3G, H, J, and K), consistent with reduced structural stability and a conformation more amenable to trigger-induced opening. This structural destabilization primarily enhanced ON-state expression (Fig. 3B, available as supplementary data at *Bioinformatics* online), while leaky expression remained largely unaffected (Fig. 3C, available as supplementary data at *Bioinformatics* online), thereby driving the overall increase in switching efficacy. This trend was corroborated by experimental data, in which the mean ON–OFF (ON-state expression—OFF-state leakage) score decreased with increasing G content and increased with increasing U content in this window (Figs 3F and I, and 5C). However, the relationship between MFE and performance was non-monotonic, indicating that optimal switch performance was constrained within an intermediate energetic regime (Fig. 4C).

**Figure 4:**
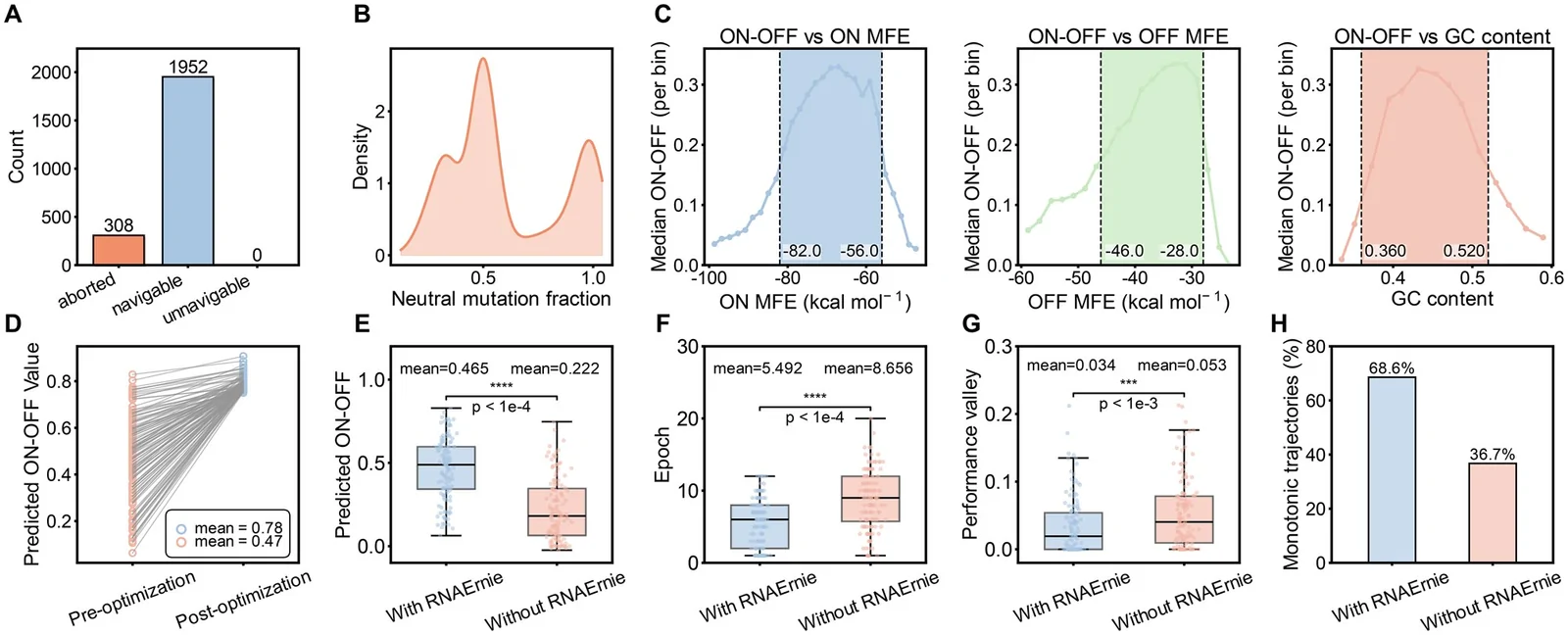
Efficient RNAErnie-guided optimization of RNA toehold switches. (A) Threshold navigability of toehold switch dataset under the task-agnostic random fitness landscape ($F_{p}$). Among 2260 randomly selected starting genotypes, 1952 (86.4%) had at least one navigable path where terminal genotype corresponds to a phenotype in the high-fitness set $H_{\tau }$ (Eq. (21)). The remaining 308 trials reached the computational budget before classification, and no completed trial was classified as unnavigable. Because these aborted trials may still contain threshold-navigable paths that were not discovered within the computational budget, the observed 86.4% fraction should be interpreted as a heuristic- and budget-dependent lower bound on the true navigable fraction. (B) Neutral mutation steps constitute a substantial fraction of navigable trajectories under the task-agnostic random fitness landscape. The mean neutral mutation fraction is 60.22%, supporting a substantial role for neutrality in maintaining navigability in the RNA switch landscape. (C) High-performing switches are concentrated within restricted ranges of ON-state MFE, OFF-state MFE, and GC content, which were used to define biophysical constraints for optimization. Shaded regions indicate the resulting constraint ranges. Curves show the median ON–OFF values obtained from equal-width bins of ON-state MFE, OFF-state MFE, or GC content, respectively. Constraint ranges were defined as the contiguous high-performance plateau containing the peak bin, with bin medians at least 50% of the peak median. (D) Predicted 128 ON–OFF values before and after optimization. Optimization shifts the distribution toward higher predicted ON–OFF values, with the mean increasing from 0.47 to 0.78. (E–H) RNAErnie guidance improves candidate quality and latent-space optimization efficiency (blue, with RNAErnie guidance; orange, without RNAErnie guidance; all *P* values were calculated using a two-sided Mann–Whitney *U* test). (E) Distribution of predicted ON–OFF values for generated RNA switch sequences with or without RNAErnie guidance. RNAErnie-guided generation shifts the distribution toward higher predicted switch performance, indicating that the model preferentially proposes higher-quality starting candidates. (F) Optimization epochs required to reach the top-5% performance threshold of the reported dataset from the same 128 initial sequences. RNAErnie-guided optimization reaches this threshold in fewer rounds, indicating improved latent space search efficiency. (G) Distribution of performance valley depths across the 128 optimization trajectories. RNAErnie-guided trajectories exhibit shallower valleys, indicating reduced detours through low-performance regions during optimization. (H) Fraction of monotonically improving trajectories among the 128 trials. RNAErnie guidance increased the proportion of trajectories exhibiting non-decreasing predicted ON–OFF performance across successive optimization steps.

**Figure 5:**
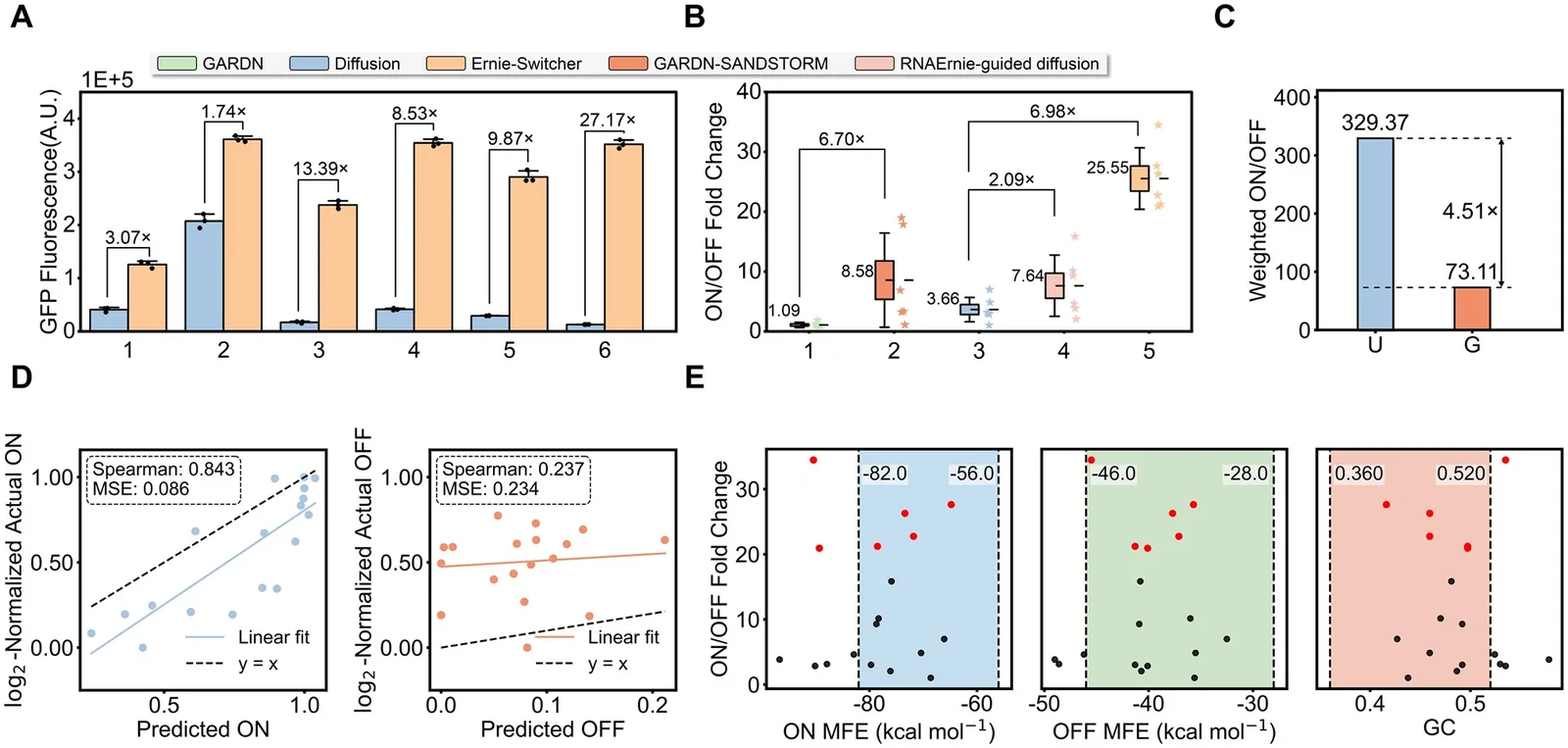
RNAErnie-guided design improves *in vivo* performance through accurate prediction guidance and biophysical constraints. (A) ON-state GFP fluorescence in *E. coli* for 12 RNA toehold switches (six generated by Diffusion and six by Ernie-Switcher). Ernie-Switcher increased ON-state expression by an average of 10.63-fold relative to Diffusion (one-sided Wilcoxon rank-sum test, P<  $1\times 10^{-8}$; three biological replicates per construct). (B) Experimental ON/OFF scores of 30 synthetic RNA toehold switches in *E. coli*, comprising six sequences generated by each of five design methods (center lines of boxes denote means; each star represents the measured performance of one switch). (C) Experimental support for the design rule identified by sequence analysis. Weighted ON/OFF scores [computed by Eqs. (14) and (15)] were higher for U than for G (329.37 versus 73.11), indicating that enrichment of U and depletion of G in the last three nucleotides of the recognition region are associated with improved switch performance (as identified in Fig. 3E). (D) Prediction performance for ON-state expression and OFF-state leakage. Predicted ON-state values showed strong agreement with experimental measurements (Spearman correlation = 0.843; MSE = 0.086), whereas OFF-state predictions showed weak rank agreement (Spearman correlation = 0.237; MSE = 0.234). (E) Consistency of experimentally tested designs with the predefined biophysical constraint windows. Each point represents one of 18 experimentally tested switch sequences (six designs per strategy: diffusion, RNAErnie-guided diffusion and Ernie-Switcher in panel B). Red points denote the six highest-performing sequences. Eighty-three percent (15/18) of the design-constraint pairs fall within the constraint windows, indicating that the high-performing experimental designs largely recapitulated the biophysical patterns identified from the training data (Fig. 4C) and complied with the constraints imposed during optimization.

Together, these findings illustrate that deep learning-based predictions can accelerate the identification of high-performance RNA toehold switches through *in silico* screening, and can also help resolve sequence–structure–function relationships into experimentally testable design principles. By uncovering and validating such rules, this framework provides a basis for the rational design and optimization of RNA toehold switches.

### 3.4 Efficient RNAErnie-guided optimization of RNA toehold switches

The intrinsic navigability of a GP landscape provides a theoretical foundation for the feasibility of sequence optimization. Therefore, we first investigated whether the RNA toehold switch dataset exhibits this topological property. To this end, we systematically characterized the threshold navigability of the RNA toehold switch dataset under task-agnostic landscapes (see the Section 2.9 for evaluation method). Under the task-agnostic random fitness landscape, the observed lower bound on the threshold-navigable fraction was 86.4% (1952/2260) among the randomly selected starting genotypes (Fig. 4A). Despite being a heuristic- and budget-dependent estimate, this high lower bound provides evidence for substantial threshold navigability within the analyzed RNA landscape (Greenbury *et al.* 2022). Sensitivity analysis across different fitness thresholds and computational budgets showed quantitative variation in the estimated navigable fraction, while consistently indicating extensive connectivity within the sampled genotype space (Table 1, available as supplementary data at *Bioinformatics* online). Analysis of the identified navigable trajectories further showed that neutral mutations accounted for 60.22% of all mutational steps (Fig. 4B), indicating extensive neutral connectivity in the RNA switch landscape. Such connectivity may facilitate the exploration of genotype space with limited structural disruption and is consistent with previous observations that molecular sequence spaces often harbor abundant neutral or near-neutral mutations (Kimura 1977, Hughes 2008). Together, the substantial threshold navigability observed under the task-agnostic random fitness landscape supports the theoretical plausibility of RNA sequence optimization.

We next sought to identify biophysical constraints that could restrict the search space to regions enriched for high-performing switches while maintaining biological plausibility. Analysis of standardized regression coefficients identified GC content, OFF-state MFE, and ON-state MFE as the three primary determinants of performance (|β| = 0.340, 0.231, 0.227 for GC, OFF MFE, ON MFE in Fig. 5, available as supplementary data at *Bioinformatics* online). Correlation analysis further revealed that high-performing switches were enriched within specific windows of these variables (Fig. 4C). These enriched windows were subsequently defined as biophysical constraints (ON MFE: [−82, −56] kcal/mol; OFF MFE: [−46, −28] kcal/mol; GC content: [36%, 52%]). We then incorporated these constraints into the RNAErnie-guided optimization framework to improve search efficiency (Fig. 1D; see Section 2.4 for details). Under predictor guidance and the defined biophysical constraints, the mean predicted ON–OFF score increased from 0.47 before optimization to 0.78 after optimization (Fig. 4D). These findings demonstrate that high-performing RNA toehold switches remain accessible within biophysically plausible regions of sequence space. Building on the observed optimization results, we next evaluated how RNAErnie guidance contributes to the optimization process. We analyzed the optimization process from the dual perspectives of initial starting point quality and trajectory properties. Regarding the starting points, RNAErnie-guided generation significantly shifted the initial sequence distribution toward higher predicted ON–OFF values (Fig. 4E). These initial sequences also showed greater adherence to the empirically defined biophysical intervals for GC content, ON-state MFE, and OFF-state MFE (Fig. 6, available as supplementary data at *Bioinformatics* online). Together, these results indicate that RNAErnie preferentially samples starting points that are more consistent with the empirically defined biophysical intervals and exhibit higher predicted performance, thereby improving optimization efficiency. Regarding the optimization trajectories, when starting from the same set of 128 initial genotypes, RNAErnie-guided optimization reached the high-performance set in significantly fewer epochs (Fig. 4F) and traversed shallower performance troughs (Fig. 4G). Importantly, RNAErnie yielded a higher fraction of monotonically improving optimization trajectories (Fig. 4H). Together, these findings indicate that RNAErnie improves RNA switch optimization by providing higher-quality starting candidates and enabling faster and more stable latent space search.

**Figure 6:**
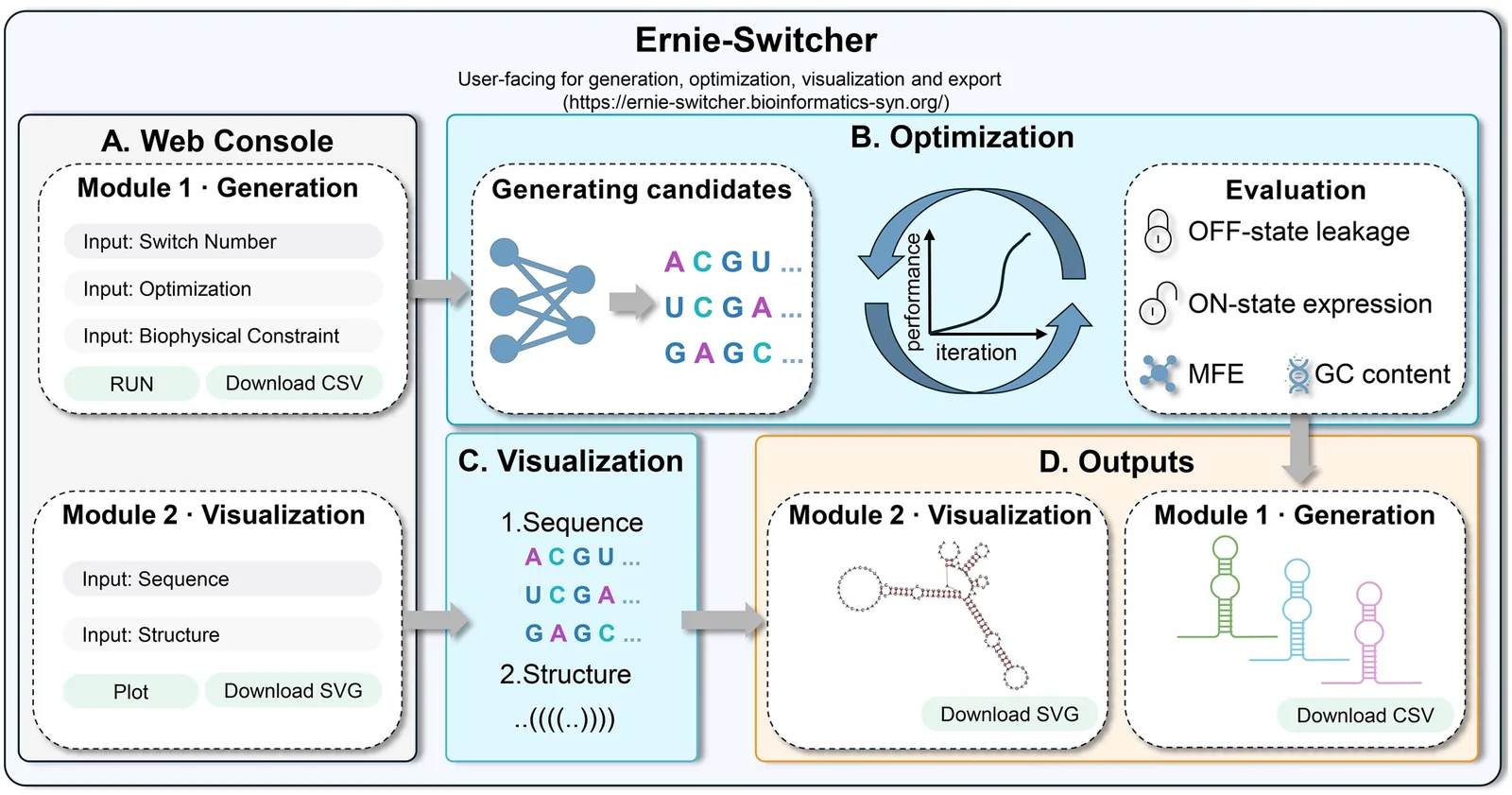
Overview of the Ernie-Switcher platform for RNA toehold switch design. (A) The web console comprises two modules for RNA toehold switch generation and RNA secondary structure visualization. (B) Optimization workflow in Module 1. Candidate sequences are generated from user-defined parameters (e.g. batch size, biophysical constraints) and iteratively refined through evaluation of OFF-state leakage, ON-state expression, MFE, and GC content. (C) Visualization workflow in Module 2. This module renders RNA secondary structures from sequence and dot-bracket inputs via ViennaRNA (Lorenz *et al.* 2011). (D) Output panel showing downloadable design results, including candidate sequence libraries (.csv format) and RNA secondary structure visualizations (.svg format).

### 3.5 Experimental validation of designed high-performance switches in *E. coli*

To validate the biological functionality of the designed switches, we performed *in vivo* experiments in *E. coli*. We initially asked whether Ernie-Switcher improved ON-state output and overall switching efficacy. Consistent with the computational predictions, all six optimized switches exhibited markedly higher ON-state expression, with GFP output increasing by an average of 10.63-fold relative to the Diffusion designs (Fig. 5A). This enhancement was accompanied by superior switching performance, with Ernie-Switcher achieving the highest mean ON/OFF score of 25.55 and the lead candidate attaining an ON/OFF score of 35 (Fig. 5B). This advantage reflected two synergistic effects. Even without predictor-feedback optimization, RNAErnie-guided sequence generation yielded higher ON/OFF scores than Diffusion and GARDN, confirming that RNAErnie-derived representations effectively biased designs toward high-fitness regions of the sequence space. With predictor-feedback optimization, Ernie-Switcher increased the mean ON/OFF ratio from 3.66 to 25.55, corresponding to a 6.98-fold improvement. In comparison, GARDN-SANDSTORM increased the mean ON/OFF ratio from 1.09 to 8.58, corresponding to a 6.70-fold improvement. Although the relative fold improvements were broadly comparable, Ernie-Switcher achieved a substantially greater absolute increase in ON/OFF performance, supporting the effectiveness and improved optimization capability of our framework.

To ascertain whether these *in vivo* advantages were consistent with the sequence, predictive, and biophysical signatures inferred during computational design, we performed a detailed characterization of the synthesized constructs. In agreement with the sequence rule inferred from mutational analysis (Fig. 3E), the aggregate weighted contribution score within the terminal 3-nt window of the recognition region was substantially higher for U than for G (329.37 versus 73.11 in Fig. 5C). Subsequently, predicted ON-state expression showed strong concordance with experimental measurements (Spearman = 0.843 in Fig. 5D), whereas OFF-state predictions showed a weak correlation (Spearman = 0.237 in Fig. 5D), indicating that predictor-informed scoring provided partial guidance for sequence optimization, while its ability to capture OFF-state leakage requires further validation. In parallel, across the six highest-performing constructs, 15 of 18 (83.33%) design-constraint pairs (three constraints per design: ON-state MFE, OFF-state MFE, and GC content) fell within the inferred windows (Fig. 5E). This concordance suggests that the empirically derived biophysical windows provided useful guidance for selecting experimentally high-performing candidates, while the biological mechanisms underlying these associations remain to be elucidated. Together, these results indicated that the *in vivo* gains achieved by Ernie-Switcher remained consistent with the sequence patterns, predictive behavior, and biophysical constraints inferred from the computational framework.

### 3.6 A web-based platform for customized RNA toehold switch design

To facilitate the broader dissemination and practical utility of our framework, we developed Ernie-Switcher, a web-based platform for automated RNA toehold switch design (Fig. 6). The platform is built around a core generation module that empowers users to configure critical design parameters, including the number of candidate switches, optional biophysical constraints, and whether closed-loop optimization is applied. In a single execution cycle, Ernie-Switcher is capable of generating and evaluating up to 32 candidate sequences within approximately five minutes. For each candidate, the platform provides a comprehensive performance profile, encompassing ON-state and OFF-state MFE, predicted ON-state expression, predicted OFF-state leakage, and GC content. The platform also includes a visualization module for rendering RNA secondary structures from either generated sequences or user-provided inputs, enabling rapid inspection of candidate designs. Collectively, this intuitive interface bridges the gap between sophisticated computational modeling and laboratory application, providing the broader biological community with a rigorous yet user-friendly tool for precision RNA engineering.

## 4 Discussion

In this study, we presented Ernie-Switcher, a deep learning framework that leverages RNAErnie to develop an interpretable design system for engineering high-performance programmable RNA devices. We validated this framework on RNA toehold switches. In *E. coli* validation assays, the best-designed RNA toehold switch achieved a 35-fold ON/OFF ratio (Fig. 5B).

A central contribution of this work is the repurposing of an RNA foundation model from a general representation learner into an interpretable engineering system. On the generative front, incorporating RNAErnie embeddings as sequence-structure priors increased the similarity of generated switches to reported functional sequences at both the sequence and structural levels, without compromising diversity (Fig. 2D and E). Corroborating this, RNAErnie guidance nearly doubled the mean ON/OFF performance in biological assessments (from 3.6-fold for the diffusion baseline to 7.6-fold for RNAErnie-guided diffusion in Fig. 5B). On the prediction front, we fused RNAErnie embeddings with primary sequence, secondary structure, and dimer interaction, which allowed the predictor to leverage both local motif signals and global structural context. As a result, our predictor showed the best agreement with experimental measurements (Spearman = 0.813 and 0.642 in Fig. 3A and C), thereby providing a more reliable ranking signal for design prioritization. From an engineering perspective, the synergy between guided generation and robust ranking streamlines the design-build-test-learn (DBTL) cycle by filtering out candidates with limited experimental potential, thereby improving the efficiency of experimental resource allocation.

Computational analyses further indicate that landscape navigability provides a theoretical basis for sequence optimization, whereas RNAErnie contributes practical improvements in candidate quality and optimization efficiency. The observed lower bound on threshold navigability (86.4% in Fig. 4A and Table 1, available as supplementary data at *Bioinformatics* online) indicates that monotonic single-nucleotide paths to high-fitness regions can be identified for many sampled starting sequences, thereby supporting the theoretical feasibility of RNA switch optimization. Separately, RNAErnie captures sequence–structure relationships that improve candidate generation and latent space optimization. RNAErnie-guided generation produces higher scoring starting candidates with greater consistency with the empirical biophysical constraints, while subsequent optimization reaches the predefined performance threshold more rapidly and with shallower performance troughs. Together, these complementary components support a more informed and efficient RNA design workflow i.e. compatible with iterative laboratory and automated design pipelines.

Beyond model-derived guidance, we incorporated empirically defined biophysical constraints to restrict the search space toward sequence regions enriched for high-performing switches. MFE and GC content were selected because of their established relevance to RNA folding and sequence composition, whereas the specific constraint ranges were derived empirically from the training data. These constraints were therefore used to guide optimization toward empirically enriched and biophysically plausible sequence regions (Fig. 5E).

Several limitations should be acknowledged. The mechanistic relationships between the imposed biophysical constraints and high-performance switching remain incompletely understood. In addition, the limited predictive accuracy for the OFF state, together with the use of a single predicted secondary structure to represent each sequence, may incompletely capture trigger binding and the dynamic conformational transition between the OFF and ON states, thereby limiting the fidelity of functional evaluation. A further limitation is that the estimated threshold navigability depends on the selected fitness threshold and available computational resources. Limited search budgets may miss existing navigable paths, leading to an underestimation of the true navigable fraction.

In summary, Ernie-Switcher provides not only a performance improvement for RNA toehold switch design, but also a more usable engineering workflow for programmable RNA device development. More broadly, the practical significance of this framework lies in its potential to make programmable RNA device development more efficient, more predictable, and more accessible. A framework that improves first-pass candidate quality and supports iterative optimization could reduce screening burden and accelerate the translation of programmable RNA parts into practical settings. While further validation across diverse device classes remains necessary, the overarching strategy of combining foundation model priors, navigability analysis, and biophysical grounding provides a robust blueprint for engineering complex nucleic acid systems, including ribozymes and aptazymes.

## Supplementary Material

btag774_Supplementary_Data
