# Supplementary material for: Ernie-Switcher: RNA foundation model-guided design of programmable RNA devices in a navigable landscape
Source: Bioinformatics. 2026 Oct 26;42(11):btag774. doi: 10.1093/bioinformatics/btag774 (PMC13726693; doi:10.1093/bioinformatics/btag774)
Supplement: Supplementary file 1 [file btag774_supplementary_data.zip › Supplementary Figures and Tables.pdf]

Supplementary Figures and Tables for paper: "*Ernie-Switcher: RNA foundation model-guided design of programmable RNA devices in a navigable landscape*"

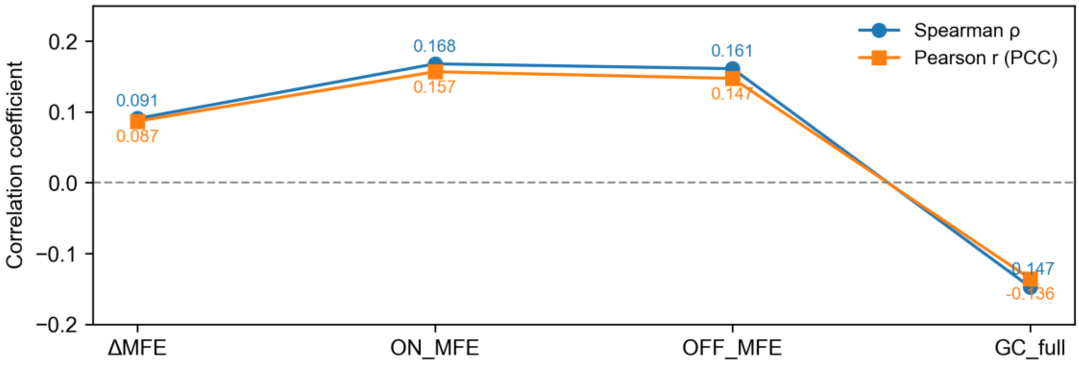


**Figure. S1 Correlation analysis between key features and switch performance.** Spearman's rank correlation (ρ) and Pearson's correlation (PCC) between the switch performance (ON-OFF) metric and key features (ΔMFE= ON_MFE - OFF_MFE, ON_MFE, OFF_MFE and GC_full) in the Toehold mRNA dataset. The grey dashed line indicates r/ρ=0. Overall correlations cluster near zero, indicating that a small set of simple features is insufficient to explain ON-OFF variability and motivating higher-capacity modelling to capture richer sequence/structure determinants.

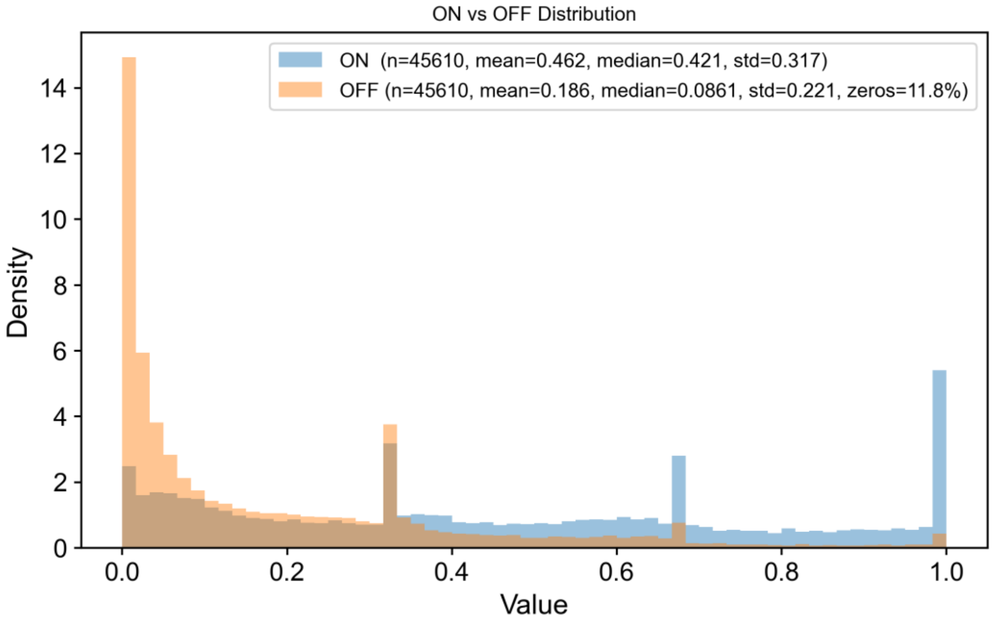


**Figure. S2 Distributions of ON and OFF labels in the toehold switch dataset.** Overlaid density histograms of ON (blue) and OFF (orange) measurements for 45,610 sequences. OFF values are strongly concentrated near 0 (including ~11.8% exact zeros) with a compressed dynamic range, which reduces effective variability and makes rank-ordering less reliable.

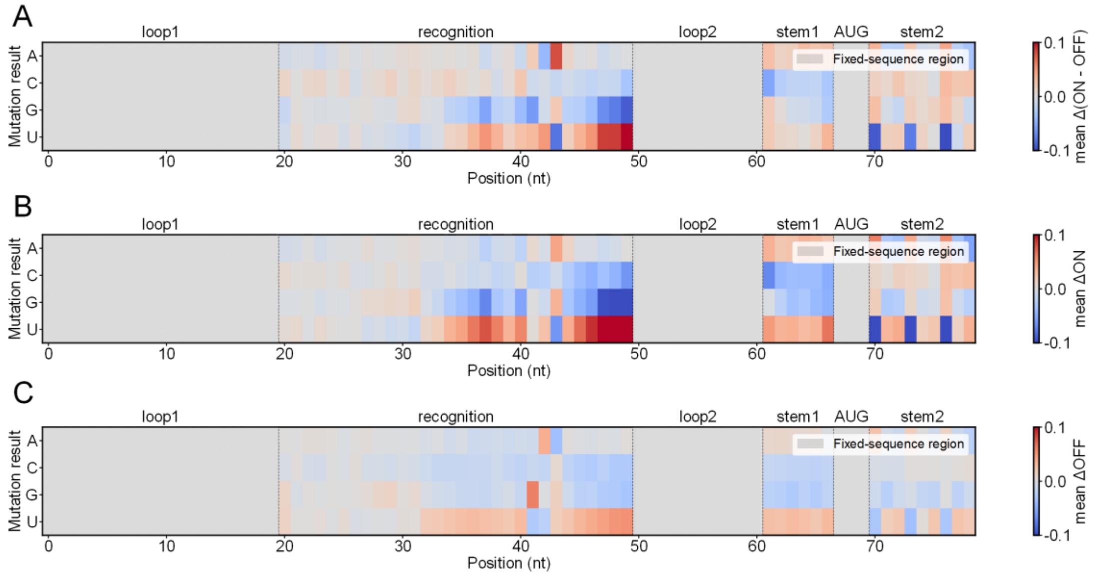


**Figure. S3 Mean effect maps of single-nucleotide saturation mutagenesis across the RNA toehold switch sequence.** Heatmaps report the mean predicted score change for each substitution at each position. Dashed vertical lines denote annotated regions (loop1, recognition, loop2, stem1, AUG, stem2). (A) Predicted switch score (ON-OFF). (B) Predicted OFF-state score. (C) Predicted ON-state score.

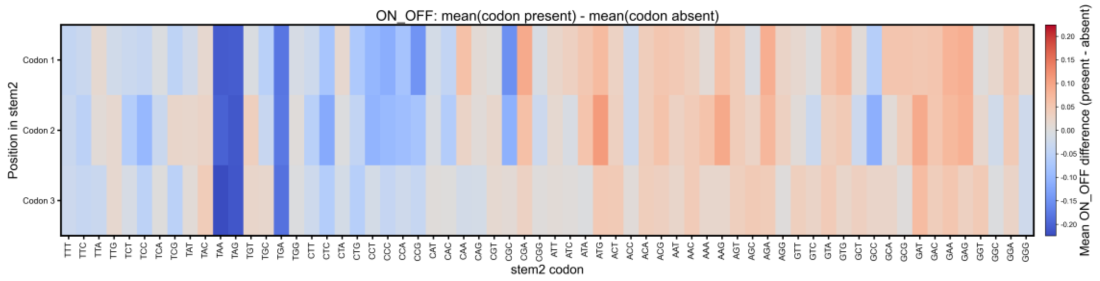


**Figure. S4 Position-specific associations between Stem2 codon identity and RNA switch ON−OFF performance.** The 9-nt Stem2 sequence was divided into three consecutive codons. Rows represent the first, second and third codon positions in Stem2, and columns represent the 64 possible codons. For each codon at each position, the heatmap shows the difference between the mean ON−OFF value of switches containing that codon and the mean ON−OFF value of switches containing any of the other 63 codons at the same position. Red indicates an association with higher ON−OFF performance, whereas blue indicates an association with lower ON−OFF performance. This descriptive analysis included 45,610 switches with valid 9-nt Stem2 sequences and complete ON, OFF, and ON−OFF measurements. The three stop codons showed a clear association with reduced ON−OFF performance, consistent with disruption of reporter translation, whereas the biological significance of the remaining codon-specific patterns requires further investigation.

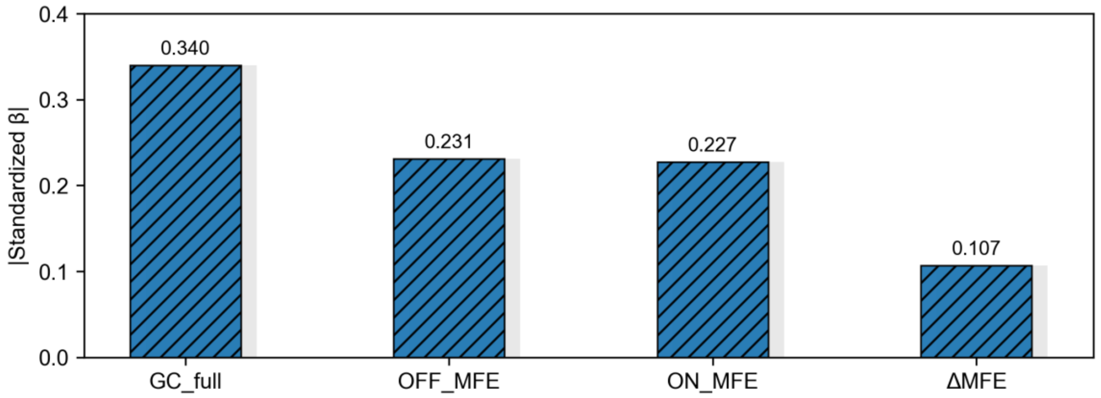


**Figure. S5 Standardized regression coefficients from a multivariate linear model highlight relative linear contributions to ON-OFF.** Fitted ordinary least squares regression after z-score standardization of all features (MFE, ON_MFE, OFF_MFE and GC_full) and the response (ON-OFF), reporting absolute standardized coefficients (|β|). Bar heights indicate each feature's relative linear contribution to ON-OFF conditional on the other variables (numbers above bars denote |β|). This analysis enables practical prioritization of constraint selection by favouring predictors with larger |β| as biologically/physically grounded constraints during sequence exploration.

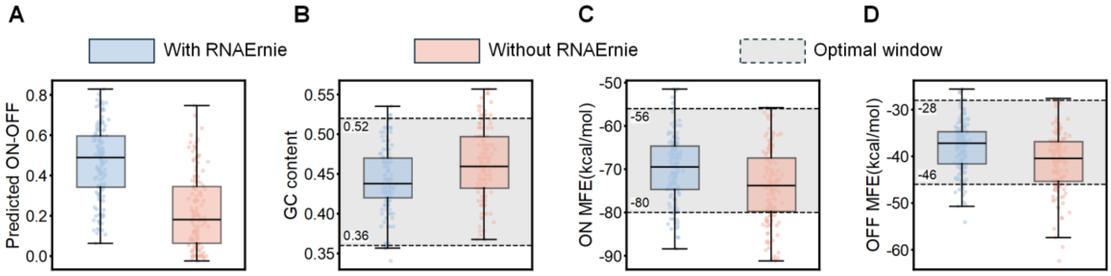


**Figure. S6 RNAErnie guidance improves candidate quality and biophysical feasibility at initialization.** All statistics are computed from 128 sequences independently generated by diffusion models with or without RNAErnie guidance. (A) Distribution of predicted ON–OFF values for sequences generated by diffusion models with and without RNAErnie guidance. Initialization with RNAErnie produces sequences with substantially higher ON–OFF values, indicating a more favorable starting point for downstream optimization. (B–D) Distributions of GC content (panel B), ON-state minimum free energy (ON MFE, panel C), and OFF-state minimum free energy (OFF MFE, panel D). The dashed regions indicate empirically defined optimal windows. Sequences generated with RNAErnie are more concentrated within these feasible regions, demonstrating improved compliance with biophysical constraints at initialization.

**Table. S1 Threshold navigability outcomes across nine combinations of fitness threshold and computational budget.** Threshold navigability was assessed under nine combinations of fitness threshold (85th, 90th, and 95th percentiles) and computational budget ($10^6$, $10^7$, and $10^8$ visited genotypes per starting sequence). The observed navigable fraction ranged from 80.97% to 100.00% across these conditions. Higher computational budgets were associated with increased recovery of threshold-navigable paths, whereas more stringent fitness thresholds reduced the observed navigable fraction, indicating a clear dependence of the quantitative estimate on both search parameters. No starting genotype was formally classified as unnavigable; trials in which no qualifying path was identified within the specified computational budget were classified as budget-exhausted. Under the primary setting used in the main analysis (95th-percentile fitness threshold and a budget of $10^8$ visited genotypes), 1,952 of 2,260 starting genotypes were classified as navigable, corresponding to an observed navigable fraction of 86.37%, while the remaining 308 trials were budget-exhausted. Because these 2,260 starting genotypes constitute only a minute sample of the full $4^{45}$ sequence space, the observed fractions should be interpreted as sample- and budget-dependent estimates rather than as direct measures of global navigability. **Nevertheless, the consistently high recovery of threshold-navigable paths across all nine conditions indicates pronounced local accessibility and connectivity within the sampled high-dimensional genotype space. (**All computational analyses were performed on a server equipped with two Intel Xeon Platinum 8380 CPUs, providing 80 physical cores and 160 logical threads in total, and the complete set of computations required approximately seven days under this hardware configuration.**)**

| Threshold | Computational Budget | Navigable | Budget-Exhausted | Unnavigable | Navigable Fraction (%) |
|---|---|---|---|---|---|
| 85% | $1\times10^6$ | 2238 | 22 | 0 | 99.03% |
| 85% | $1\times10^7$ | 2256 | 4 | 0 | 99.82% |
| 85% | $1\times10^8$ | 2260 | 0 | 0 | 100.00% |
| 90% | $1\times10^6$ | 2112 | 148 | 0 | 93.45% |
| 90% | $1\times10^7$ | 2131 | 129 | 0 | 94.29% |
| 90% | $1\times10^8$ | 2157 | 103 | 0 | 95.44% |
| 95% | $1\times10^6$ | 1830 | 430 | 0 | 80.97% |
| 95% | $1\times10^7$ | 1915 | 345 | 0 | 84.73% |
| 95% | $1\times10^8$ | 1952 | 308 | 0 | 86.37% |
